# The diagnosis of anti-LGI1 encephalitis varies with the type of immunodetection assay and sample examined

**DOI:** 10.3389/fimmu.2022.1069368

**Published:** 2022-12-15

**Authors:** Guillermo Muñoz-Sánchez, Jesús Planagumà, Laura Naranjo, Rocío Couso, Lidia Sabater, Mar Guasp, Eugenia Martínez-Hernández, Francesc Graus, Josep Dalmau, Raquel Ruiz-García

**Affiliations:** ^1^ Immunology Department, Centre Diagnòstic Biomèdic, Hospital Clínic Barcelona, Barcelona, Spain; ^2^ Neuroimmunology Program, Institut d’Investigacions Biomèdiques August Pi i Sunyer (IDIBAPS), Barcelona, Spain; ^3^ Neurology Department, Hospital Clínic, and University of Barcelona, Barcelona, Spain; ^4^ Centro de Investigación Biomédica en Red, Enfermedades Raras (CIBERER), Madrid, Spain; ^5^ Neurology Department, University of Pennsylvania, Philadelphia, PA, United States; ^6^ Catalan Institution of Research and Advanced Studies (ICREA), Barcelona, Spain

**Keywords:** neuronal antibodies, Lgi1, diagnostic test, autoimmune encephalitis (AE), immunofluorescent assay

## Abstract

Detection of Leucine-rich glioma inactivated 1 (LGI1) antibodies in patients with suspected autoimmune encephalitis is important for diagnostic confirmation and prompt implementation of immunomodulatory treatment. However, the clinical laboratory diagnosis can be challenging. Previous reports have suggested that the type of test and patient’s sample (serum or CSF) have different clinical performances, however, there are no studies comparing different diagnostic tests on paired serum/CSF samples of patients with anti-LGI1 encephalitis. Here, we assessed the clinical performance of a commercial and an *in house* indirect immunofluorescent cell based assays (IIF-CBA) using paired serum/CSF of 70 patients with suspected anti-LGI1 encephalitis and positive rat brain indirect immunohistochemistry (IIHC). We found that all (100%) patients had CSF antibodies when the *in house* IIF-CBA was used, but only 88 (83%) were positive if the commercial test was used. In contrast, sera positivity rate was higher with the commercial test (94%) than with the *in house* assay (86%). If both serum and CSF were examined with the commercial IIFA-CBA, 69/70 (98.5%) patients were positive in at least one of the samples. These findings are clinically important for centers in which rat brain IIHC and *in house* IIFA-CBA are not available. Moreover, the observation that all patients with anti-LGI1 encephalitis have antibodies in CSF is in line with the concept that these antibodies are pathogenic.

## Highlights

All patients with anti-LGI1 encephalitis harbor LGI1 antibodies in CSF. These antibodies are detectable by indirect immunofluorescence cell based assay (IIF-CBA) on cells co-expressing LGI1 and ADAM23.

In patients with suspected autoimmune encephalitis, both serum and CSF should be examined if a commercial IIF-CBA is used for antibody determination.

## Introduction

Autoimmune Encephalitis constitutes a group of inflammatory brain diseases that are characterized by prominent neuropsychiatric symptoms associated with autoantibodies against neuronal cell-surface proteins, ion channels or neurotransmitter receptors (1). Anti-LGI1 associated encephalitis has an estimated annual incidence of 0.83 per 1 million persons, and it represents the most common cause of autoimmune encephalitis of adults older than 40 years (2). Patients usually develop difficulty in forming new memories, confusion, or generalized seizures, often preceded by subtle focal seizures or faciobrachial dystonic seizures (2). Substantial cognitive response to steroids and immunotherapy occur in about 70% of patients at 24 months follow up (3). Despite the good functional status, long-term follow-up shows that 65% patients have mild cognitive impairment (4).

Leucine-rich glioma inactivated 1 (LGI1) is a neuronal secreted synaptic linker protein that interacts with the presynaptic disintegrin and metalloproteinase domain-containing protein 23 (ADAM23) and with the postsynaptic protein ADAM22, that are associated with Kv1.1 voltage-gated potassium channels and AMPA receptors, respectively (5, 6). Anti-LGI1 antibodies prevent the binding of LGI1 to ADAM23 and ADAM22, decreasing the synaptic levels of Kv1.1 and AMPA receptors and promoting neuronal hyperexcitability with increased glutamatergic transmission (6).

Anti-LGI1 antibody detection is important for the diagnosis of the encephalitis and prompt treatment with immunotherapy (7). Most clinical laboratories use the same commercial cell-based indirect immunofluorescence assay on transfected cells with LGI1 anchored to the cell membrane (Autoimmune Encephalitis Mosaic 6 kit; Euroimmun, Lübeck Germany). Several studies have reported that when using CSF, a negative result was found in 37%-47% of patients with anti-LGI1 encephalitis (2, 8, 9). These studies suggested that anti-LGI1 encephalitis, unlike other encephalitis mediated by antibodies against neuronal surface antigens, can occur without antibodies in CSF, and that the sensitivity of antibody detection is superior in serum than CSF. Contrary to this concept, our preliminary experience using more comprehensive techniques suggests that most of these patients have antibodies in CSF, and some do not have antibodies detectable in serum (10). Here, we examined in a large cohort of patients with anti-LGI1 encephalitis the performance of two indirect immunofluorescent cell-based assays (IIF-CBA) in which the LGI1 protein is expressed differently on the cell surface. The findings are important because suggest that current clinical testing misses the diagnosis in some patients.

## Materials and methods

### Patients and samples

We retrospectively examined 70 consecutive patients with available paired serum and CSF known to be LGI1 antibody positive by IIHC in at least one of the samples and a clinical suspicion of autoimmune encephalitis according to Graus et al. (7). All 140 samples were tested by 1) rat brain tissue indirect immunohistochemistry, 2) commercial cell-based IIF (Autoimmune Encephalitis Mosaic 6 kit; Euroimmun, Lübeck Germany) in which cells are only transfected with LGI1 and 3) *in-house* cell-based IIF in which cells are co-transfected with LGI1 and one of its natural synaptic ligands (ADAM23). All studies were examined by two independent observers.

### Rat brain indirect immunohistochemistry

Rat brain tissue indirect immunohistochemistry was performed in all CSF and sera as previously described (11). Briefly, adult Wistar rats were euthanized in a CO2 chamber and the brain was removed without previous tissue perfusion. Brains were sagittally split in two hemispheres, immersed in 4% paraformaldehyde for 1h at 4°C, cryoprotected with 40% sucrose for 48h, and snap frozen in chilled isopentane. Frozen sections were air-dried for 30 min and sequentially treated with hydrogen peroxide 30% in PBS for 15 minutes. Brain sections were blocked with 5% normal goat serum in PBS for 1h at room temperature and incubated with patients’ sera (diluted 1:200) or CSF (1:2) overnight at 4°C. Biotinylated goat anti-human IgG (Vector Labs, Burlingame, CA, USA) was added for 2 h, followed by incubation with the avidin–biotin immunoperoxidase complex (Vector Labs, Burlingame, CA, 114 USA) for 1 h. The reaction was developed with 0.05% diaminobenzidine (Sigma, St. Louis, MO, USA). Animal studies were approved in accordance with European (2010/63/UE) and Spanish (RD 53/2013) regulations by the ethics committee of Hospital Clínic of Barcelona.

### Indirect immunofluorescence cell-based assays

All samples were subsequently examined with two types of IIF-CBA: 1) a commercial assay, Autoimmune Encephalitis Mosaic 6 kit (Euroimmun, Lübeck Germany), following manufacturer’s instructions and recommended dilutions (undiluted CSF and 1:10 serum), to test IgG antibodies against N-methyl-D-aspartate (NMDA) receptor (GluN1), a-amino-3-hydroxyl-5-methyl-4- isoxazole-propionate (AMPA) receptor (GluA1, GluA2), gamma-aminobutyric (GABA) B receptor (B1 and B2 subunits), contactin-associated protein-like 2 (CASPR2), leucine-rich glioma-inactivated protein 1 (LGI1) and dipeptidyl-peptidase 6 (DPPX), and 2) in-house IIF-CBA in which Human Embryonic Kidney 293 (HEK293) cells were transfected with DNA constructs to express LGI1 together with ADAM23 (12). Briefly, sera and CSF were diluted in PBS-1% BSA (1:2 CSF and 1:10 serum) and incubated with stored pre-fixed transfected cells overnight. Immunodetection was performed using an goat anti-human IgG antibody conjugated with AF488 (A11013, Invitrogen,Waltham, MA, USA) for 1 h. IIF-CBA results were observed in an Axio-Imager 2 microscope (Carl Zeiss, Jena, Germany).

The study was approved by the ethics committee of Hospital Clínic of Barcelona. Patients’ samples were coded and clinical information was anonymized prior to analysis.

## Results

### Study cohort

The median age of the patients was 62 years (range, 26-83 years), and 27 (40%) were women (no demographic information available from 3 patients). From the 66 patients with available information, the most common clinical findings were seizures (70%), followed by cognitive impairment (62%), memory loss (59%) and behavioral changes (38%) (**Box 1**. Representative Case). Brain MRI studies showed abnormalities compatible with limbic encephalitis in 38 out of 49 (78%) patients. EEG was abnormal in 27 out of 38 (71%) patients. CSF pleocytosis was found in 4 out of 41 (10%) patients. One patient had ovarian teratoma and another patient had invasive thymoma. Two patients had concurrent neuronal surface antibodies (one against the GABA_B_ receptor and another against CASPR2).

### LGI1 antibody detection

Among the 70 sera examined, 69 (98.5%) were positive by brain indirect immunohistochemistry, 66 (94%) by commercial IIF-CBA, and 60 (86%) by *in-house* IIF-CBA. Regarding the 70 paired CSF samples, 68 (97%) were positive by brain indirect immunohistochemistry, 58 (83%) by commercial IIF-CBA, and 70 (100%) by *in-house* IIF-CBA.

47 (67%) patients were positive for LGI1 antibodies in both samples (CSF and serum) with the three indicated techniques, brain immunohistochemistry, commercial IIF, and *in-house* IIF (*concordant patients*). The other 23 (33%) patients showed discordant findings *(discordant patients)* that resulted from the technique employed (commercial vs *in-house* assay) or the sample tested (serum or CSF) (**Table 1**). A comprehensive analysis of the results from discordant patients showed that the commercial assay missed LGI1 antibodies in CSF more frequently than in serum (12 [52%] vs. 4 [17%]), and one patient was negative in both serum and CSF. In contrast, the *in-house* assay missed LGI1 antibodies in serum more frequently than in CSF (10 [43%] vs. 0 [0%]). All samples negative by one of the assays were positive by the other. No clinical differences were found between concordant and discordant groups of patients (**Supplementary Table 1**). Patients’ samples were negative by IIF-CBA with HEK293 cells expressing only ADAM23 (not shown).

**Table 1:** Findings in serum and CSF of patients with anti-LGI1 encephalitis and discordant results in the indicated tests.

	Brain IIHC		Commercial IIF-CBA (LGI1 expressed in HEK293 cells)		*In-House* IIF-CBA (LGI1/ADAM23 expressed in HEK293 cells)	
Discordant Patient	CSF	Serum	CSF	Serum	CSF	Serum
1	+	+	+	+	+	–
2	+	+	+	+	+	–
3	+	+	+	+	+	–
4	+	+	+	+	+	–
5	+	+	+	+	+	–
6	+	+	+	+	+	–
7	+	+	+	+	+	–
8	+	+	–	+	+	+
9	+	+	–	+	+	+
10	+	+	–	+	+	+
11	+	+	–	+	+	+
12	+	+	–	+	+	+
13	+	+	–	+	+	+
14	+	+	–	+	+	+
15	+	+	+	–	+	+
16	+	+	+	–	+	+
17	+	+	+	–	+	+
18	+	+	–	+	+	–
19	+	+	–	+	+	–
20	–	+	–	+	+	–
21	–	+	–	+	+	+
22	+	–	+	+	+	+
23	+	+	–	–	+	+

IIHC, Indirect Immunohistochemistry; CSF, Cerebrospinal Fluid; IIF-CBA, immunofluorescence cell-based assay using HEK293 cells expressing LGI1. In the commercial assay LGI1 is expressed on the cell surface with a transmembrane domain, whereas in the in house assay the LGI1 is co-expressed with disintegrin and metalloproteinase domain-containing protein 23 (LGI1/ADAM23).

**Box 1 Representative case.**

**Figure 1:**
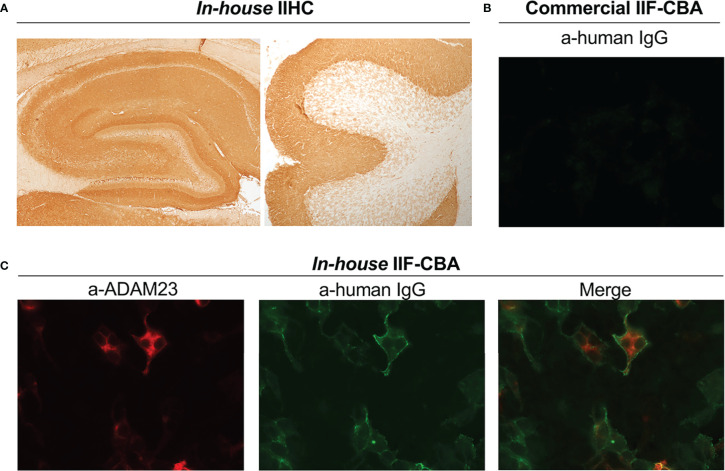
LGI1 antibody detection using different tests. **(A)** Patient’s CSF showing LGI1 immunoreactivity with rat brain tissue (IIHC), including hippocampus (left) and cerebellum (right). **(B)**Image showing the lack of reactivity of the patient’s CSF with HEK293 cells expressing LGI1(commercial indirect immunofluorescent cell based assay) **(C)** In-house IIF-CBA show the reactivity of the same sample with HEK293 cells coexpressing LGI1 and ADAM23: Left, expression of ADAM23 confirmed with a commercial antibody (a-ADAM23); Middle, reactivity of patient’s antibodies with LGI1; Right, merge. Patient’s CSF did not react with HEK293 cells expressing only ADAM23 (not shown). IIF-CBA: indirect immunofluorescent cell based assay; ADAM23: disintegrin and metalloproteinase domain-containing protein 23.

A 62-year-old woman developed confusion, dizziness and rapid cognitive decline over 3 weeks. Symptoms substantially worsened during the last week and her family described strange behavior and episodes of aggression. Two days before admission to the hospital, she developed faciobrachial dystonic seizures (3-4 per day). At admission, the MRI showed increased FLAIR/T2 signal predominantly involving the left hippocampus, and the EEG showed slow activity. Blood chemistry was normal except for hyponatraemia (128 mEq/L), and the CSF showed mild pleocytosis with 7 leukocytes/mm^3^. CSF testing using the Autoimmune Encephalitis Mosaic 6 kit (Euroimmun) was negative for all antibodies (NMDA receptor, AMPA receptor, GABA-B receptor, CASPR2, LGI1 and DPPX). Given the suspicion for autoimmune encephalitis, a sample of CSF was sent to our center for further analysis. The CSF sample was tested by rat brain IIHC, showing robust immunostaining of the hippocampus in a pattern highly suggestive of LGI1 antibodies (**Figure 1A**). However, the same CSF sample was negative in the commercial assay (**Figure 1B**). Subsequently, CSF and serum were tested by *in-house* IIF-CBA, confirming the presence of LGI1 antibodies in both samples (**Figure 1C**).

## Discussion

In this study, we show that using an IIF-CBA co-expressing LGI1 and ADAM23 all patients with LGI1 autoimmune encephalitis had LGI1 antibodies in CSF. Thus, the concept that in this type of autoimmune encephalitis the antibodies predominantly occur in serum should be reconsidered. The reason why one IIF-CBA performs better than the other depending on the type of sample (serum or CSF) is unclear. We postulate that the repertoires of antibodies in serum and CSF are different and that when using CSF, the expression of LGI1 with its natural ligand facilitates the detection of antibodies. Irrespective of these or other unsuspected reasons the findings have important diagnostic implications.

Previous studies suggested that up to 47% of patients with anti-LGI1 encephalitis only have detectable LGI1 antibodies in serum (2, 8, 9) (**Table 2**). These studies used the IIF-CBA technique that expresses LGI1 anchored to the cell membrane instead of the IIF-CBA that co-expresses LGI1 with ADAM23. In addition, most studies do not use brain immunohistochemistry with CSF, which unambiguously shows intensive immunostaining in a pattern characteristic of LGI1.

**Table 2:** Reported results for LGI1 antibody detection in CSF by IIF-CBA.

Study	Type of IIF-CBA	LGI1 antibodies in CSF (%)
van Sonderen A, et al. (2) (Neurology, 2016)	Commercial (LGI1)	9/17(53%)
Gadoth A, et al. (8) (Ann Neurol, 2017)	Commercial (LGI1)	24/38 (63%)
Muñiz-Castrillo S, et al. (13) (Neurol Neuroimm Neuroinfl, 2021)	In-house (LGI1 co-transfected with ADAM22)	105/134 (78%)
McCracken L, et al. (9) (Neurology, 2017)	Commercial (LGI1)	4/7 (57%)
Present Study	Commercial (LGI1) in-house (co-expression of LGI1 and ADAM23)	58/70 (82%) 70/70 (100%)

The implications of our findings are not only clinical but also pathogenic, challenging the concept that the mechanism of this disease, regarding antibody synthesis, is different from most autoimmune encephalitis in which the prevalence of antibodies is higher in CSF. In autoimmune encephalitis, CSF samples represent better the immune dysregulation that takes places within the CNS than serum samples, and the importance of CSF for antibody detection has been demonstrated in most of these disorders (14). This is well illustrated in anti-NMDA receptor encephalitis where given the risk of false-negative results using serum, most investigators have adopted CSF antibody testing (15, 16). Moreover, in some autoimmune encephalitis, the clinical significance of the detection of antibodies only in serum is unclear; for example, serum GABA_A_ receptor or glycine receptor antibodies may occur in patients with a wide variety of diseases or symptoms that are not immune-mediated or do not respond to immunotherapy (17, 18).

In the present study we show that screening of any sample (CSF or sera) by IIHC and subsequent confirmation of antibodies in CSF with the IIF-CBA that co-expresses LGI1 with ADAM23 is the best approach to detect LGI1 antibodies. All patients included in our cohort (N=70) were positive with this approach. Although the commercial IIF-CBA with LGI1 alone was more sensitive in serum than our IIF-CBA expressing LGI1-ADAM23, the commercial assay performed substantially worse with CSF. Moreover, the diagnosis of 4 patients would have been missed using serum with the commercial IIF-CBA, which is the standard diagnostic test for LGI1 antibodies in most clinical laboratories. In practice, some of these cases are later diagnosed in research laboratories due to the persistence of symptoms and high level of clinical suspicion, but delays in treatment can affect outcome (3, 19, 20). Therefore, clinical laboratories using the commercial IIF-CBA as screening method, should consider including CSF testing if serum is negative and no alternative diagnosis is identified.

In our experience, brain tissue IIHC is a useful complementary test for antibody detection in autoimmune encephalitis. As shown here, this is particularly important for LGI1 antibodies because the brain tissue IIHC with either serum or CSF is highly sensitive and produces a pattern of hippocampus and cerebellum immunostaining that is very suggestive of these antibodies (12).

A task for the future is to determine the repertoire of LGI1 antibody specificities in serum and CSF. We postulate that autorreactive B cells present in CNS are in prolonged contact with the antigen and undergo somatic hypermutation and affinity maturation, facilitating the selection and expansion of auto-reactive B cell clones that specifically recognize LGI1 linked to its natural ligands. Our study suggests heterogeneity among patients’ antibodies regarding LGI1 epitope recognition and provides the best strategy to reduce false negative results with tests currently available.

## Data availability statement

The raw data supporting the conclusions of this article will be made available by the authors, without undue reservation.

## Ethics statement

The studies involving human participants were reviewed and approved by the ethics committee of Hospital Clínic of Barcelona. Patients’ samples were coded and clinical information was anonymized prior to analysis. Written informed consent for participation was not required for this study in accordance with the national legislation and the institutional requirements. The animal study was reviewed and approved in accordance with European (2010/63/UE) and Spanish (RD 53/2013) regulations by the ethics committee of Hospital Clínic of Barcelona.

## Author contributions

JD, RR-G, EM-H, JP, GM-S and FG designed the study and wrote the manuscript. RR-G, JP, GM-S, LN, LS and RC performed laboratory work and/or data analysis. JP, EM-H and MG collected clinical information from the LGI-1 patients. All authors contributed to the article and approved the submitted version.
