# Supplementary material for: The diagnosis of anti-LGI1 encephalitis varies with the type of immunodetection assay and sample examined
Source: Front Immunol. 2022 Dec 15;13:1069368. doi: 10.3389/fimmu.2022.1069368 (PMC9798107; doi:10.3389/fimmu.2022.1069368)
Supplement: Supplementary file 1 [file Table_1.docx]

**Supplementary Table 1.** Neurological manifestations of patients with anti-LGI1 encephalitis according to tests results

|  | **Discordant (%)** |  | **Concordant (%)** |  | ***P* value** |
| --- | --- | --- | --- | --- | --- |
| Seizures | 14/22 (64) |  | 32/43 (74) |  | 0,3658 |
| Memory loss | 15/22 (68) |  | 24/43(56) |  | 0,3355 |
| Cognitive Impairment | 14/22 (64) |  | 27/43 (63) |  | 0,9467 |
| Behavioral changes | 6/22 (27) |  | 19/43 (44) |  | 0,1848 |
| CSF Pleocytosis | 4/17 (24) |  | 1/17 (4) |  | 0,0620 |
| MRI abnormalities | 14/18 (78) |  | 24/28 (86) |  | 0,4883 |
| EEG activity altered | 12/16 (75) |  | 15/22 (68) |  | 0,6473 |

Discordant, indicates patients with LGI1 antibodies detected with only some tests; Concordant indicates patients with LGI1 antibodies detected with all the tests used.
